# Retraction Note: Qilongtian ameliorate bleomycin-induced pulmonary fibrosis in mice via inhibiting IL-17 signal pathway

**DOI:** 10.1038/s41598-023-38495-x

**Published:** 2023-07-12

**Authors:** Qiang Zhang, Ting Luo, Dezheng Yuan, Jing Liu, Yi Fu, Jiali Yuan

**Affiliations:** 1 School of Basic Medicine, Shanghai University of Traditional Chinese Medicine, 1200 Cailun Road, Pudong District, Shanghai, 201203 China; 2 Yunnan Provincial Key Laboratory of Molecular Biology for Sinomedicine, Yunnan University of Chinese Medicine, Kunming, 650500 China; 3 Yunnan University of Chinese Medicine, Kunming, 650500 China; 4 The Third Affiliated Hospital of Yunnan University of Chinese Medicine: Kunming Municipal Hospital of Traditional Chinese Medicine, Kunming, 650500 China

Retraction of: *Scientific Reports* https://doi.org/10.1038/S41598-023-31439-5, published online 12 April 2023

The Authors have retracted this Article because it has been published previously by some of the same Authors^1^. This Article is therefore redundant.

All Authors agree to this retraction.
